# Integrins, cadherins and channels in cartilage mechanotransduction: perspectives for future regeneration strategies

**DOI:** 10.1017/erm.2021.16

**Published:** 2021-10-27

**Authors:** Martin Philipp Dieterle, Ayman Husari, Bernd Rolauffs, Thorsten Steinberg, Pascal Tomakidi

**Affiliations:** 1 Division of Oral Biotechnology, Center for Dental Medicine, Medical Center – University of Freiburg, Faculty of Medicine, University of Freiburg, Hugstetter Str. 55, 79106 Freiburg, Germany; 2 Department of Orthodontics, Center for Dental Medicine, Medical Center – University of Freiburg, Faculty of Medicine, University of Freiburg, Hugstetter Str. 55, 79106 Freiburg, Germany; 3 Department of Orthopedics and Trauma Surgery, G.E.R.N. Research Center for Tissue Replacement, Regeneration & Neogenesis, Medical Center – Albert-Ludwigs-University of Freiburg, Faculty of Medicine, Albert-Ludwigs-University of Freiburg, 79085 Freiburg im Breisgau, Germany

**Keywords:** Cadherins, cartilage, cell instruction, channels, integrins, intervertebral disc, matrix, mechanotransduction, osteoarthritis, regeneration

## Abstract

Articular cartilage consists of hyaline cartilage, is a major constituent of the human musculoskeletal system and has critical functions in frictionless joint movement and articular homoeostasis. Osteoarthritis (OA) is an inflammatory disease of articular cartilage, which promotes joint degeneration. Although it affects millions of people, there are no satisfying therapies that address this disease at the molecular level. Therefore, tissue regeneration approaches aim at modifying chondrocyte biology to mitigate the consequences of OA. This requires appropriate biochemical and biophysical stimulation of cells. Regarding the latter, mechanotransduction of chondrocytes and their precursor cells has become increasingly important over the last few decades. Mechanotransduction is the transformation of external biophysical stimuli into intracellular biochemical signals, involving sensor molecules at the cell surface and intracellular signalling molecules, so-called mechano-sensors and -transducers. These signalling events determine cell behaviour. Mechanotransducing ion channels and gap junctions additionally govern chondrocyte physiology. It is of great scientific and medical interest to induce a specific cell behaviour by controlling these mechanotransduction pathways and to translate this knowledge into regenerative clinical therapies. This review therefore focuses on the mechanotransduction properties of integrins, cadherins and ion channels in cartilaginous tissues to provide perspectives for cartilage regeneration.

## Introduction

### Introduction about cartilaginous tissues: articular hyaline cartilage and fibrocartilage of the intervertebral disc

Cartilaginous tissues are important constituents of the human musculoskeletal system, because they are indispensable for joint movement, shock absorption and the distribution of compressive loading. Histologically, articular cartilage, fibrocartilage and elastic cartilage are distinguished.

Elastic cartilage can be found in the ear conch or the epiglottis and is, from a biochemical point of view, comparable with hyaline cartilage (see below). It additionally comprises elastic fibres, which render it resistant to bending. Elastic cartilage is not further discussed herein.

Fibrocartilage unifies the mechanical properties of hyaline cartilage, namely compressive strength, with the tensile strength of connective tissue. In addition to collagen type II, it also harbours collagen type I, which is normally found in skin or bone. The annulus fibrosus of the intervertebral disc (IVD) consists of fibrocartilage (see below).

The most common type of cartilage, hyaline cartilage, is an integral component of joints and is important for frictionless articulation, resistance to compressive loading, and joint homoeostasis. It consists of chondrocytes, which are embedded in an extracellular matrix (ECM). The chondrocytes are derived from aggregated mesenchymal stem cells (MSCs) that differentiate into chondrogenic progenitor cells (CPCs) and subsequently into chondroblasts. The chondroblasts synthesise the cartilage-specific ECM. Chondrocytes do not synthesise matrix anymore. One or more chondrocytes form the chondron, which represents the functional unit of cartilage tissue (Refs 1–6). The ECM is mainly composed of collagen (type II, IX and XI in articular cartilage), and the ground substance composed of hyaluronic acid, proteoglycans (mainly aggrecan and small leucine-rich repeat proteoglycans such as decorin, biglycan, fibromodulin and lumican) and glycoproteins (e.g. cartilage oligomeric matrix glycoprotein) (Ref. 7).

Cartilage homoeostasis is a complex process arising from biochemical and biophysical signals. As biochemical factors are not the focus of this review, we direct the interested reader to our recent review (Ref. 8). Multiple biophysical factors such as the stiffness of the chondrocytes and the ECM (Refs 9–15) and additional factors such as mechanical loading, oxygen partial pressure and nutrient supply contribute to cartilage homoeostasis. Disequilibrium of these factors leads to degenerative cartilage pathologies, amongst them osteoarthritis (OA) or IVD degeneration (Refs 16–18). Millions of people are affected by those painful, chronic degenerative and inflammatory diseases, leading to constraints in daily activities and disability. This includes pain, immobility and the incapacity for work (Refs 19–21). A recent study estimated the global prevalence of knee OA to be around 16.0% in people aged 15 years and over (Ref. 22). In 2013, healthcare costs for OA exceeded $16 billion in the USA (Ref. 23).

The pathophysiology of OA, which affects hyaline articular cartilage, is complex and incompletely understood. The main steps can be summarised as follows: aetiologic (OA secondary to trauma, ischaemia or metabolic) and unknown factors (primary OA) induce a cascade of cartilage destruction. Enzymes such as the matrix-degrading matrix metalloproteinases (MMPs, especially MMP-9, -13, -16 and -28) lead to the catabolic degradation of cartilage ECM proteins. This goes along with a loss of the mechanical properties of the joints, that is, they lose their compressive strength. The destruction of the cartilage is accompanied by an inflammatory reaction. The latter involves pro-inflammatory cytokines such as interleukin-1 (IL-1) and immune cells such as macrophages, T- and B-cells, which migrate to the site of inflammation. The chondrocytes themselves also respond to these stimuli. They become hypertrophic and finally die or transdifferentiate into osteoblasts. The subchondral bone is consequently exposed to more mechanical load. Reparative adaption leads to the formation of sclerotic subchondral bone, which can be detected radiologically (Ref. 24).

IVD consists of the central nucleus pulposus (NP) and the peripheral annulus fibrosus, which consists of fibrocartilage. Similar to OA, IVD degeneration is characterised by an imbalance of matrix degradation and synthesis. Pro-inflammatory mediators such as IL-1 and tumour necrosis factor *α* (TNF-*α*) as well as MMPs promote the disease process. Nutrition, ageing, metabolic factors and genetic susceptibility contribute to the onset and progression of IVD (Ref. 25). Irrespective of the age and degeneration stage, combined mRNA and protein expression analysis in NP cells revealed the expression of characteristic NP cell markers, namely forkhead box F1, paired box protein 1, keratin (K)8 and K18, and carbonic anhydrase-12 (CA12). In addition, NP cells also showed expression of notochord cell markers such as the transcription factor brachyury, galectin-3 and CD24 in the cells of the NP (Ref. 26). This expression profile clearly distinguishes IVD cells from articular chondrocytes. Despite the similarity in OA and IVD degeneration pathophysiology, more research is needed to specify the disease-specific cellular reactions and the influence of the developmental history of each cell type on the disease process.

Clinical therapies to treat damaged articular cartilage lesions in patients prior to clinically apparent OA include autologous chondrocyte implantation (ACI), which is currently the well-established gold-standard for treating large articular cartilage defects (Refs 27–31). Other procedures rely on osteochondral transplants or combine methods such as bone grafting and ACI (Refs 32–37). Bone marrow stimulating (BMS) (Ref. 38) treatments such as microfracture, one of the most commonly performed surgical articular cartilage repair procedures, rely on the influx of MSCs from the surgically penetrated subchondral bone, to initiate (fibro-)cartilaginous repair (Refs 39–42) of small localised articular cartilage defects (Ref. 27). An emerging clinical procedure termed ‘autologous matrix-induced chondrogenesis’ (AMIC™) utilises the influx of MSCs from surgically penetrated subchondral bone, but in conjunction with administering a collagen type I/III membrane (Refs 43–47). However, BMS methods are somewhat limited by defect size and the fact that fibrocartilaginous tissue is formed upon microfracture treatment, which differs greatly from articular cartilage in its biochemical and biophysical properties and, thus, may limit repair tissue durability (Refs 48, 49). Current last resort therapies mainly focus on the removal of the dysfunctional joint tissues and on implanting joint replacement endoprostheses (Refs 50–54).

In the IVD degeneration context, current treatment strategies focus on physical exercise, pain management or surgical approaches to stabilise or decompress the affected vertebral segments (Ref. 55). However, translational MSC-based clinical approaches showed promising initial results in preclinical models and some clinical trials (Refs 56–61). In the study by Orozco *et al*., bone marrow-derived MSCs were injected into the NP in 10 patients suffering from chronic back pain. The therapy led to a sustained pain reduction (Ref. 61). A randomised, controlled trial with allogeneic MSCs by Noriega and colleagues even showed an improvement in the disc quality (Ref. 60). A comparable clinical outcome was observed in two patients following percutaneous implantation of autologous MSCs into the degenerated IVD (Ref. 62). In the context of tissue engineering, cell-loaded *in situ* photopolymerisable poly(ethylene glycol) dimethacrylate nano-fibrillated cellulose composite hydrogels appeared to be promising candidates to regenerate the NP and also the total IVD. Because of its tailored mechanical properties, the hydrogel was able to withstand the mechanical needs of this IVD model. Disc height after surgery could be re-established and maintained for 0.5 million loading cycles (Ref. 63).

As can be observed from these examples, the development of targeted treatments that causally address the biophysical (dys-)functions of diseased cartilaginous tissues is of great scientific and clinical interest. Additionally, the different cell populations and developmental stage (e.g. stem cell versus more differentiated stage) of the corresponding tissues need to be considered (Refs 64–74). In this context, a basic understanding of the physiological and pathophysiological biomechanical properties and mechanosignalling pathways in cartilaginous tissues is helpful. Thus, after having discussed the basic principles of cartilage biology, the consequences of ECM-derived biophysical stimuli on chondrocytes are introduced next.

### ECM-derived biophysical cues

Mechanotransduction can be defined as the molecular signalling cascade, which transforms an extracellular physical stimulus into an intracellular biochemical signal. Consequently, mechanical stimulation influences cell behaviour, including cell functions such as proliferation and differentiation (Refs 75–78). Compression or shear forces are typical physical stimuli that can be sensed by cells, including chondrocytes (Refs 79–81). These forces are needed to maintain a mechanotransduction-dependent tissue homoeostasis at the cellular and molecular levels under physiological conditions. Thus, mechanical stimulation has been used in many experimental studies to induce cartilage-specific mRNA expression, as indicated by the expression of for example, collagen II, aggrecan, Sry-box transcription factor 9 (Sox9) and cartilage oligomeric matrix protein (Refs 82–89). To make these behavioural decisions, cells need to sense and integrate biophysical signals originating from their environment, the latter represented by either the ECM, neighbouring cells or synthetic substrates (Refs 90–95). Molecules that sense and compute such biophysical signals are part of cell-to-cell or cell-to-matrix interactions and are in most cases directly or indirectly linked to the intracellular actin filament system (Refs 96–98). In the case of cell–matrix interactions in cartilage, integrin-containing focal adhesions (FAs) mediate mechanotransduction (Refs 99–103). FAs are complex, three-dimensional (3D) molecular clutches with important roles in differentiation and cell migration. They are discussed in detail in Section ‘Mechanotransduction at the cell–matrix interface’. Conversely, cell-to-cell contacts in the early phase of the articular chondrocyte life cycle and also in the NP contain neural (N)-cadherins (Refs 104–112). Apart from supporting mesenchymal cell condensation, cadherin-based junctions act as important signalling platforms for the integration of mechanobiological information from different cellular pathways. Section ‘Mechanotransduction at the cell–cell interface’ is dedicated to their function. Ion channel-associated mechanotransduction (Section ‘Channel-associated mechanosignalling’) and changes in the actin cytoskeleton (Section ‘The role of the actin cytoskeleton in chondrogenesis’) are also the consequences of ECM-derived biophysical cues.

Prospective strategies for inducing tissue regeneration should therefore trigger these pathways in a regeneration-supporting way. This requires an exact knowledge of the distinct natural environmental parameters within cartilage, including both biochemical and biophysical factors. Regarding the biophysical cues, ECM stiffness (elasticity) (Refs 113–119), topographical ECM characteristics (Ref. 120) and the spatial patterns of the microenvironment (Refs 121–124) as well as cell adhesion points (Refs 125–127), have been shown to influence cell behaviour. Therefore, the ECM-derived biophysical information is one key factor to understand cartilage biology. It must be considered when designing biomaterials for cartilage regeneration purposes. Therefore, the next section focuses on how to make such materials ‘cell instructive’.

### Biomaterials and the concept of cell instruction

Regenerative cartilage biology tries to imitate the in vivo processes of cartilage formation and maintenance. The overall goal is the construction of mechanically resistant materials that enable the generation of the right type of cartilage in the right place.

Several biomaterials for cartilage tissue regeneration are currently clinically available. These are membranes consisting of collagen types I and III, on which patient-derived cells are seeded for ACI. Hyaluronan- or/and albumin-hyaluronic acid hydrogels for ACI have also been described. Additionally, cell-free biomaterials for microfracture/AMIC have been developed. Such membranes consist of collagen type I or collagen types I and III (Refs 128, 129).

Bio-Seed^®^-C (BioTissue Technologies, Freiburg, Germany) is a representative example for a fully synthetic biomaterial used in cartilage regeneration. It is a porous 3D scaffold made of polyglycolic acid, polylactic acid and polydioxanone that has been seeded with autologous chondrocytes embedded within a fibrin gel (Ref. 130). Bio-Seed^®^-C has been reported to induce the formation of articular cartilage, which is associated with a significant clinical improvement in joint function. Such synthetic polymers have a delayed degradation rate. They can be combined with natural polymers, that is, elastin, fibrin and collagen, and thereby exhibit a synergistic effect for various tissue regeneration applications (Ref. 131).

With respect to synthetic hydrogels, Bian and co-workers could show that methacrylated hyaluronic acid -based hydrogels support the chondrogenesis of hMSCs. By employing these hyaluronic acid hydrogels, which were biofunctionalised with N-cadherin mimetic peptides as well as epitopes for the interaction with CD44 and CD168 (both hyaluronic acid receptors), they demonstrated early chondrogenesis in conjunction with cartilage-specific matrix synthesis during long-term culture. Neocartilage formation in an in vivo nude mouse model was also enhanced (Ref. 132).

In the broader context of biomaterials, synthetic glycosaminoglycan (GAG)-mimetic networks, for instance composed of hydrophilic zwitterionic monomers such as 2-methacryloyloxyethyl phosphorylcholine for hydration and the cross-linker ethylene glycol dimethacrylate for network formation (Ref. 133), offer possibilities for articular cartilage augmentation and regeneration. This is because these hydrogels can be cross-linked *in situ* to augment OA-dependent loss of cartilage-specific GAGs. This stiffens the cartilage matrix and restores its function. Such *in situ* photo-cross-linked hydrogels, also called interpenetrating polymer networks (IPNs), help to reconstitute the mechanical properties of degraded articular cartilage (Ref. 134).

Natural polymers represent a biocompatible alternative with superior biological and cell interactive properties (Ref. 135). A platform based on distinct and mutable mechanical properties has already been developed by using (VPGVG)*_n_* (Val-Pro-Gly-Val-Gly amino acid motif) elastin- and recombinant resilin materials. It can be used to readily introduce mutations into pentapeptide sequences, thereby modulating biophysical properties (Refs 136–141). Focused on articular cartilage regeneration, the elastic modulus, reflected by a natural polymer-based biomaterial platform, should be around 0.2–1.0 MPa (Ref. 136). Further current trends in biofabrication of biomaterials, their modifications and biophysical stimulations for cartilage regeneration, are reviewed by Przekora and Kelly (Refs 142, 143).

The above-described regenerative approaches are only a small selection of innovative strategies to treat cartilage pathologies. However, they have one major shortcoming: although they try to mimic the mechanical properties (or the biochemical composition) of the cartilage ECM, they do not specifically address the tissue-inherent mechanotransduction pathways of the chondrocytes. Thus, the OA or IVD degeneration-induced imbalances are not actually treated. The cells and/or their environment is/are simply replaced or supported by an artificial matrix.

However, the ideal therapeutic biomaterial-based strategy would be a medical device, which includes and supports tissue-specific biomechanical properties. This includes a cellular environment that triggers signalling pathways relevant to regeneration. One way to approach this goal is to construct bio-inspired materials, that is, materials whose mechanical and biochemical properties resort to naturally occurring molecules.

By incorporating the elasticity of the cell-surrounding ECM in conjunction with the nano-patterning of adhesion-relevant ECM ligands, materials that closely resemble the cell's physiological environment can be designed. In the context of chondrocyte biology, this means that, for example, cartilage defects are repaired with the help of a biomaterial that mimics the ECM of chondrocytes. Thus, naturally occurring signalling pathways are triggered by that material, which eventually and ideally leads to a complete healing of the defect. Such materials are termed cell-instructive, as they literally accompany and guide the cells in their environment on the way to becoming a functional tissue in the proper localisation. This is because the biomaterial contains the biophysical and biochemical information for the cells to adhere, spread, proliferate or differentiate similar to under in vivo or developmental conditions. Conventional biomaterials are mainly used as simple delivery vehicles for cells or drugs (non-instructive). Cell-instructive materials, however, actively control the tissue functions through determining as many relevant parameters as possible (e.g. cell adhesion points, migratory stimuli, material stiffness, stimulation of signalling pathways, spatiotemporally adjusted release of pharmacological compounds, etc.) (Ref. 144).

The concept of cell instruction can exemplarily be explained by gel-in-gel hydrogels (Ref. 145). This is a class of water-binding, polymeric biomaterials that have been intensively studied in the context of tissue engineering. They create both permissive and instructive conditions for the survival, growth, assembly and differentiation of cells. They are either composed of (i) reconstituted assemblies of natural ECM constituents, (ii) synthetic components, for instance poly(ethylene glycol) (PEG ) or (iii) semisynthetic biohybrid materials such as GAG-containing materials. Advances in the design of engineered hydrogel systems were achieved by the systematic tuning of biophysical and biomolecular features. This includes the modulation of the gel layering, coating, the introduction of spatial material gradients, fibre production and assembly, compartmentalisation of the material and many other properties (Ref. 145).

Furthermore, simple chemical methods have been developed that allow for coupling of nearly any functional group to hydrogel polymers to enable specific cell stimulation in tissue regeneration. This strategy is exemplified by an approach of Guo and colleagues, who developed a modular hydrogel cross-linker, poly(glycolic acid)–poly(ethylene glycol)–poly(glycolic acid)-di(but-2-yne-1,4-dithiol) (PdBT), which can be biofunctionalised by tissue-specific biomolecules. In the cartilage context, biofunctionalisation was carried out with N-cadherin as well as the cartilage-related GAG chondroitin sulphate. To design a biodegradable hydrogel, poly(*N*-isopropylacrylamide) was used as a polymer, which was spontaneously cross-linked by the above-mentioned biomolecule-functionalised PdBT, to allow for successful MSC encapsulation (Ref. 146).

Another interesting approach, which addresses both repair of cartilage and the subchondral bone was published by Kang *et al*. This approach is based on a poly(ethylene glycol)-diacrylate and *N*-acryloyl 6-aminocaproic acid triphasic hydrogel, which underwent covalent cross-linking through radical polymerisation. The tri-layered scaffold included a calcium phosphate-biomineralised bottom layer for the stimulation of bone formation, a cryogel middle layer with anisotropic pore architecture, and a hydrogel top layer for chondrogenesis. By co-culturing MSCs and chondrocytes, the chondrocyte hypertrophy, which is typical of OA, could be avoided. Within the middle and the top layers, the authors could achieve MSC chondrogenic differentiation and cartilage formation in vitro. Subcutaneous implantation of the pre-cultured chondro-constructs led to the recruitment of host cells to the biomineralised bottom layer with subsequent bone tissue formation. This tri-layered scaffold, therefore, harbours the properties of a cell-instructive biomaterial. The experiments impressively show that hydrogels can be the basis for complex tissue regeneration applications. They can be fabricated with different compartments, which support the desired cell differentiation and the interaction of different tissues such as cartilage and bone (see also Section ‘Perspectives in cartilage regeneration’). Of interest, molecular processes associated with the interaction of the biomaterial and cells, for example, the activation of mechanosignalling pathways, can be studied by employing such materials. This contributes to a better understanding of the physiology and pathophysiology of cartilaginous tissues (Ref. 147).

Thus, cell instructive parameters, which address specific features of cell behaviour through mechanosignalling, are important issues of current biomaterial research. Relevant findings in the context of integrin, cadherin and ion channel signalling of chondrocytes are, therefore, cursorily depicted below.

## Mechanotransduction at the cell–matrix interface

### Integrins and their role in cartilage mechanotransduction

**Fig. 1.**
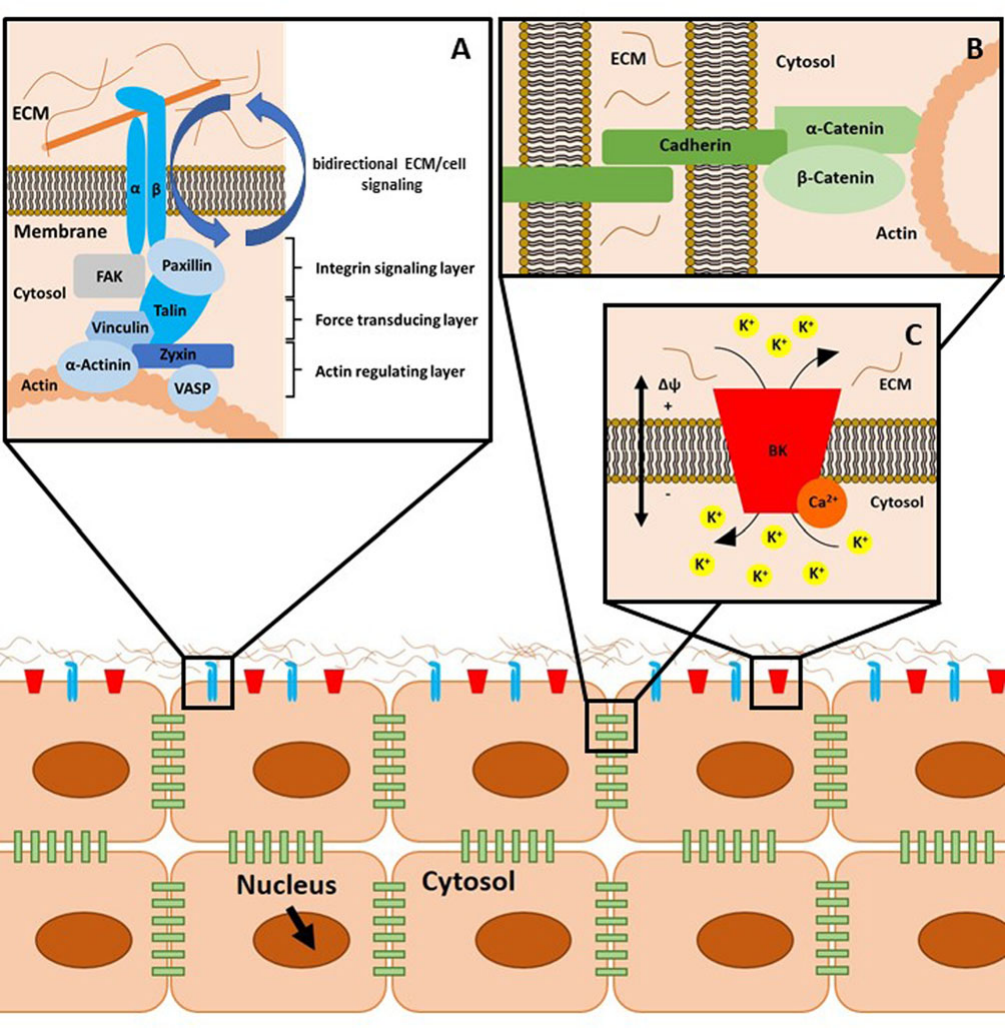
Schematic representation of cellular mechanotransduction. (a) Focal adhesions (FAs) mediate cell-to-matrix contacts. Integrins, consisting of an *α* and *β* subunit, bind their substrates, for example, collagen, extracellularly. There are three layers of adaptor and signalling proteins associated with integrins, as described in the text. Of note, FAs as well as adherens junctions (b) and ion channel-related signalling hubs (c) are supramolecular aggregates of the depicted proteins. This aspect is omitted for reasons of clarity. (b) Adherens junctions comprise cadherins, *α*-catenin and *β*-catenin and mediate cell-to-cell contacts. (c) Calcium-activated large potassium K^+^ channels (big potassium, BK) can be modulated by intracellular binding of Ca^2+^ ions. The gating behaviour of these channels is regulated by ligand binding, mechanical stimulation and by the current membrane potential Δ*ϕ*. ECM, extracellular matrix.

Integrins are membrane-embedded, heterodimeric protein complexes, which are composed of an *α* and a *β* subunit. These *α* and *β* subunits bind extracellular ECM proteins. Healthy adult articular chondrocytes express various integrins (summarised in Table 1), which all have different ligands and regulate cartilage cell behaviour. Integrins constitute the bidirectional signalling hubs of FAs. In more detail, FAs are nano-scaled structures consisting of proteins stratified in an axis perpendicular to the cell membrane (Fig. 1a). Adjacent to the plasma membrane, paxillin, focal adhesion kinase (FAK), cellular sarcoma (c-Src, herein Src), kindlin 1 and the talin head domain (N-terminus) co-localise with integrin cytoplasmic tails inside the integrin signalling layer. Talin (C-terminus) and vinculin build the intermediate force transduction layer, whereas actin binding proteins (>50 nm beneath the plasma membrane), including vinculin, zyxin, *α*-actinin and vasodilatator-stimulated phosphoprotein, in conjunction with actin, form the actin regulatory layer (Refs 101, 148–152).

**Table 1.** Integrins and their ligands in healthy and diseased cartilage

Integrins in healthy cartilage	*α*1*β*1	*α*3*β*1	*α*5*β*1	*α*10*β*1	*α*ν*β*1	*α*ν*β*3	*α*ν*β*5
Ligands	Collagens	Laminins	Fibronectin	Collagen-II, -IV, -VI	Fibronectin	Fibronectin	Vitronectin
Integrins in diseased cartilage	*α*2*β*1	*α*4*β*1	*α*6*β*1				
Ligands	Collagens	Fibronectin, V-CAM	Laminins				

Compression or shear forces in cartilaginous tissues lead to outside-in signalling, which delivers important environmental information into the cell. At the same time, FAs transduce internal signals, that is, cytoskeletal tension, to the matrix, which is called inside-out signalling. This process is crucial for cell–ECM communication (Ref. 153). It has been demonstrated in vitro that physiological amounts of mechanical stimulation of human chondrocytes increases the expression of aggrecan, and decreases MMP-3 gene expression in a pathway involving the *α*5*β*1 integrin together with an IL-4 release (Refs 154–159). Thus, a certain degree of mechanical load is needed for proper cartilage homoeostasis.

Recently, experimental findings by Woltersdorf *et al*. have, however, challenged the overall view that simple collagen–integrin interactions are crucial for chondrocyte mechanotransduction. It could be shown that binding of certain integrins to collagens is mainly limited to single collagen molecules and that interaction with non-collagenous proteins is more important for the actual mechanical function (Ref. 160).

Indirect evidence of integrin function in healthy chondrocytes also arises from manipulation of the expression of specific integrins. *β*1-integrin-deficient chondrocytes have an abnormal shape, fail to arrange into columns in the growth plate of developing bones and exhibit decreased proliferation because of defects in the cell cycle (Ref. 161). Knockout of *α*1 integrin in mice leads to an OA-like phenotype with the increased levels of MMP-2 and MMP-3 in conjunction with low levels of proteoglycans, indicating ECM destruction (Ref. 162). Thus, it can be concluded that expression of these integrin subunits is crucial for (i) chondrogenesis and (ii) cartilage maintenance. Findings on integrin signalling in diseased cartilage are scarce. Chondrocytes derived from osteoarthritic cartilage additionally express certain integrins, as depicted in Table 1 and Refs 160, 163–173. Moreover, some integrins such as *α*V that are expressed under healthy conditions show elevated expression levels in OA chondrocytes (Ref. 174). It is still not clear, which mechanisms lead to the alterations in integrin presence in chondrocytes of OA cartilage. Existing theories include effects of growth factors and cytokines, which change the expression profiles of the chondrocytes (Refs 174–176). OA as a degenerative cartilage disorder is also characterised by a damaged cartilage matrix, comprising fibronectin fragments, which are recognised by *α*5*β*1 integrin. This binding initiates a pro-inflammatory and pro-catabolic response, which contributes to advanced matrix degradation (Ref. 175). *α*5*β*1 integrin is the only integrin whose function has been systematically studied in the context of cartilage biology and diseases. Therefore, the next section deals with this specific heterodimer.

### The ambivalent role of *α*5*β*1 integrin in chondrocyte mechano-responsiveness: cartilage maintenance versus catabolic destruction

*α*5*β*1 integrin is the receptor for the ECM protein fibronectin. *α*5*β*1 integrin has an ambivalent role in mediating chondrocyte mechanoresponsiveness, which depends on whether the amount of external mechanical stimuli/loading is physiological or not.

**Fig. 2.**
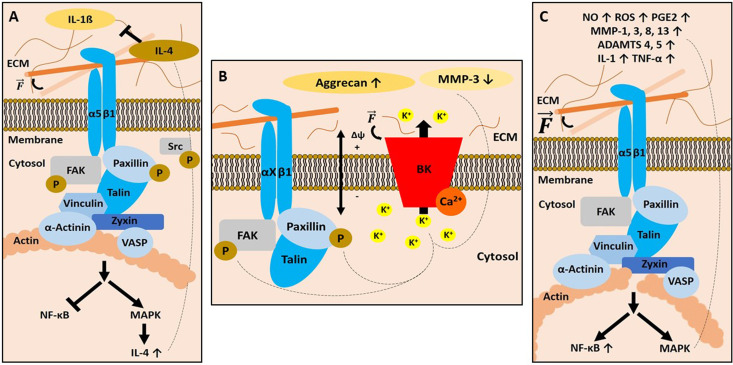
*α*5*β*1 integrin and stretch-activated ion channels (SACs) in chondrocyte biology. (a) Binding of ECM ligands (e.g. fibronectin) to *α*5*β*1 integrin leads to focal adhesion (FA) activation. Physiological amounts of mechanical loading 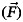 lead to phosphorylation (P) of integrin-associated signalling molecules such as focal adhesion kinase (FAK), paxillin and the kinase sarcoma (Src). These events inhibit the activation of proinflammatory pathways. Mitogen-activated protein (MAP) kinase (MAPK) activity is upregulated. This leads to the expression of the anti-inflammatory cytokine interleukin-4 (IL-4), which can block pro-inflammatory signals such as IL-1*β*. Altogether, these processes contribute to cartilage homoeostasis. (b) Mechanical loading 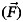 opens SACs, which is followed by a flux of ions. In the case of the calcium-activated large potassium K^+^ channels (BK), potassium ions move from the cytosol into the extracellular space. These channels are associated with FAs (denoted as *α*x*β*1 integrin to represent the interaction of various *β*1-integrin containing heterodimers with SACs). Activation of SACs also leads to phosphorylation (P) events at the FA components paxillin and FAK. At the transcriptional and protein levels, activation of BK leads to an increase in aggrecan expression and protein synthesis. Conversely, the amount of the matrix-degrading enzyme matrix metalloproteinase 3 (MMP-3) is reduced, contributing to cartilage matrix maintenance. (c) Mechanical overstimulation of FAs leads to a disruption of the actin cytoskeleton. This leads to an upregulation of nuclear factor *κ*B (NF-*κ*B), MAPK signalling and other pro-inflammatory molecules as described in the main text. ECM, extracellular matrix; Δ*ϕ*, membrane potential; K^+^, potassium ions; Ca^2+^, calcium ions.

Mechanical loading in the physiological range, that is, forces that occur during daily activities, induces signalling cascades, which lead to the production of cartilage-specific ECM components. In the presence of IL-1*β* at physiological mechanical loading, integrin-sensed mechanical stimuli inhibit nuclear translocation of the nuclear factor kappa (NF-*κ*B; p65/p50) dimers, thereby switching off transcription of pro-inflammatory genes (Ref. 177). An intact cytoskeleton is required for proper integrin mechanotransduction and mediates phosphorylation of FAK, paxillin and Src. These phosphorylation events are indirect evidence for FAs-related signalling, leading to MAP kinase activation and secretion of the anti-inflammatory cytokine IL-4 (Refs 154, 155, 158, 178–180). It is plausible that IL-4 released through an integrin-mediated mechanotransduction pathway will accumulate extracellularly and contribute to a pool of soluble anti-inflammatory mediators. These mediators block pro-inflammatory signals induced by IL-1*β* and thereby inhibit cartilage destruction (Refs 177, 181). In summary, these mechanisms are important for cartilage homoeostasis (Fig. 2a).

Mechanical overloading, that is, forces that are higher than the naturally occurring range, also activates *α*5*β*1 integrin. In this case, it leads to a disruption of the actin cytoskeletal network and stimulates members of the NF-*κ*B and MAP kinase family (Refs 182, 183). These factors increase the production of pro-inflammatory and catabolic mediators. This includes nitric oxide (NO), ECM-proteolytic enzymes such as MMP-1, -3, -8 and -13, aggrecanases (a disintegrin an metalloproteinase with a thrombospondin motif = ADAMTS) 4 and 5 (Ref. 184), reactive oxygen species (ROS), pro-inflammatory mediators such as IL-1 and TNF-*α* (Ref. 185), as well as prostaglandin PGE2 (Ref. 186) (Fig. 2c). ROS and NO are known to trigger inflammatory pathways and to oxidise various biomolecules, which impairs their function. IL-1 and TNF-*α* and their receptors are potent stimulators of the NF-*κ*B signalling axis, which supports inflammation in a positive feedback loop. The MMPs increase catabolic activities and accelerate tissue damage via the production of fibronectin (Ref. 187) or collagen fragments (Ref. 188), which bind to integrins and induce cytokines that mediate further catabolic responses (Ref. 177). It could be shown that antisense oligonucleotides directed against the *α*5 subunit of *α*5*β*1 integrin were capable of decreasing fibronectin fragment-mediated cartilage chondrolysis in bovine chondrocytes and cartilage explants (Ref. 189). This approach indirectly shows that this dimer is an important mediator of the catabolic response of cartilaginous tissues in response to unphysiological stimuli.

Altogether, these findings underscore the ambivalent role of *α*5*β*1 integrin. It serves both cell-protective and anabolic purposes under physiological mechanical loading and can also act as an ‘overload’ integrin. This means that the overstimulation of the dimer leads to a shift towards a catabolic chondrocyte metabolism, which is characteristic of OA. In addition, *α*5*β*1 integrin is involved in the mechanosensitive opening of connexin 43-built hemichannels (HCs) through direct integrin–connexin interaction (Ref. 190). This interesting facet of *α*5*β*1 integrin is discussed in more detail in Section ‘Connexin 43, gap junctions and hemichannels in chondrocyte mechanotransduction’ and depicted in Figure 2b.

## Mechanotransduction at the cell–cell interface

### Cadherins and their role in cartilage mechanotransduction

Cadherins are transmembrane proteins that are intracellularly linked to *α*-catenin, *β*-catenin and the actin cytoskeleton (Fig. 1b). Cadherin-built complexes mediate cell-to-cell adhesion and actively sense fluctuations in cellular tension or compression. This leads to tightly regulated, proportional, intracellular biochemical responses (Ref. 191).

The cadherin family member N-cadherin is predominantly expressed in mesenchymal tissues such as cartilage (Refs 97, 109, 192–194). The formation of cell-to-cell contacts is a key event during chondrogenesis and is a process called mesenchymal cell condensation/precartilage condensation. It precedes the proliferation and differentiation of chondrocytes. Homophilic cell-to-cell contacts made up from N-cadherin initiate the cell condensation of prechondrogenic mesenchymal cells, which is stabilised by neuronal cell adhesion molecule (N-CAM) later on (Ref. 195). Experiments with micromass cultures of embryonic limb mesenchymal cells have demonstrated that the N-cadherin expression level is high during active cellular condensation but decreases upon subsequent chondrogenic differentiation. This means that N-cadherin expression in mature, differentiated chondrocytes is usually low (Ref. 196). Antibody-based blocking of N-cadherin led to a significant delay in early cartilage differentiation in MSC microspheres (MMs). After establishment of cell–cell adhesions, MMs were encapsulated in collagen hydrogels (CMMs), whereas CMMs exhibited cell migration. From their results, the authors additionally concluded that the collagen hydrogel offers a suitable microenvironment for chondrogenic differentiation (Ref. 73).

In ATDC5-cells (a mouse teratoma-derived, chondrogenic cell line; see also Section ‘Rhoa and ROCK, Rac1 and Cdc42 and their role in cartilage mechanotransduction’) (Ref. 197) it was shown that the metalloproteinase a disintegrin and metalloproteinase type 10 (ADAM-10) can cleave N-cadherin extracellularly. The cleaved soluble ectodomain functions as an extracellular signalling growth factor-like molecule. Intracellularly, N-cadherin can be cleaved by the presenilin-1 (PS1) protease, leading to the dissociation of *β*-catenin, which is described below. These cleavage events lead to a switch from early cellular condensation to proliferation and are an essential step in chondrocyte differentiation (Ref. 198).

Apart from N-cadherin and the key chondrogenesis transcription factor Sox9, fibronectin and especially its integrin-binding arginine-glycine-aspartate (RGD) amino acid motif also play an important role in mesenchymal condensation (Refs 199–208). Integrin signalling and subsequent phosphorylation of FAK and its association with paxillin are key steps therein (Refs 209, 210). These findings underscore the importance of the collaboration of ECM–integrin interactions with cell-to-cell contact-forming cadherins in cartilage biology and especially in the development of cartilage tissues. Thus, mesenchymal condensation is a cooperative accomplishment of both mechanosignalling axes.

A biomaterial application of this signalling crosstalk even shed light on the downstream effectors of N-cadherin and integrin signalling during early chondrogenesis. MSCs on tunable hyaluronic acid hydrogels served as a model (Ref. 211). Cosgrove and colleagues could show that increased coupling of a hydrogel with histidine–alanine–valine–aspartic acid–isoleucine (HAVDI) adhesive sequence from the extracellular cadherin 1 (EC1) ectodomain of N-cadherin in the presence of the fibronectin-innate RGD sequence, reduced the actin-mediated cell contractility. Above, yes-associated protein (YAP) and its cellular paralogue transcriptional coactivator with PDZ-binding motif (TAZ) mechanosensing was reduced. This reduction in YAP/TAZ mechanosensing was indicated by less abundant nuclear and thus less active YAP/TAZ, which are both involved in the regulation of chondrogenesis and skeletal development (Refs 212, 213). Addition of the protease ADAM-10 to MSCs abolished the mechanotransduction-related response towards HAVDI-functionalised hydrogels by recuperation of nuclear YAP/TAZ ratios. With these experiments, the authors, amongst others, could show that HAVDI abundance alters YAP/TAZ signalling by changing the MSC mechanosensing at a given substrate stiffness (Ref. 211). In a recent study of the Bian group, the authors could show that N-cadherin peptide hydrogels suppress canonical Wnt signalling in hMSCs. In this context, it is noteworthy that Wnt signalling is dictated by *β*1-integrin-mediated and mechanotransduction-modulating ECM stiffness in primary chondrocytes (Ref. 138). The mechanism behind the suppression of Wnt signalling was substantiated by the reduction of *β*-catenin nuclear translocation. This reduction was associated with a reduced transcriptional activity of *β*-catenin, thereby enhancing the chondrogenesis of hMSCs. The authors concluded that biomimetic self-assembled peptide hydrogels can serve as a tailorable and versatile 3D culture platform to investigate the effect of biofunctionalisation on stem cell behaviour (Ref. 214).

The role of N-cadherin in adult or mature articular cartilage is less well understood. This is because to the best of our knowledge, N-cadherin has only been investigated in the context of mutational studies in OA, its expression in synovial tissues or in chondrogenesis (Refs 145, 215). Of note, synovial fibroblasts have been reported to promote OA under certain circumstances. Ruedel *et al*. reported that N-cadherin promoter polymorphisms in this cell type affect N-cadherin expression and can modulate the risk of OA development (Ref. 216). It is, therefore, plausible to conclude that N-cadherin also plays an important role in adult cartilaginous tissues, at least through the action of neighbouring cells such as NP cells and synovial fibroblasts. This may lead to an indirect role in the pathogenesis of OA and IVD degeneration.

However, there are more specific findings for NP cells concerning the function of N-cadherin. Healthy NP cells show a higher expression level of N-cadherin than cells of rats with IVD degeneration. High compression forces lead to a decrease in N-cadherin expression. Overexpression of N-cadherin in combination with compression reduces cellular apoptosis and senescence, which might be a protection from degenerative IVD diseases (Refs 217, 218).

### Impact of *β*-catenin on chondrocyte differentiation

Based on the importance of N-cadherin for chondrogenesis, it was hypothesised that the expression of N-cadherin-bound proteins such as catenins also plays a role in chondrocyte differentiation (Ref. 219). Although there are some studies that report a transcriptional role for *α*-catenin, the scientific evidence concerning its functions apart from being a junctional protein is scarce (Ref. 220). However, *β*-catenin is involved in different cellular pathways (cadherin signalling and Wnt signalling). In addition to stabilising cell–cell adhesions by interacting with N-cadherin, *β*-catenin is also engaged in the regulation of gene expression by acting as a transcriptional co-activator within the nucleus. *β*-Catenin addresses target genes such as cyclin D (Ref. 221), which are important for cell cycle progression and therefore cell proliferation. This fact defines *β*-catenin as a moonlighting protein (Ref. 222).

To fulfil its function as a transcriptional co-activator, *β*-catenin needs to detach from the cytoplasmic tail of cadherins. Another possibility of *β*-catenin translocation to the nucleus is the activation of canonical Wnt signalling, which will not be discussed in this review (Ref. 223). In the case of Wnt-independent, cadherin-based cell adhesion, *β*-catenin detachment is mediated by the PS1/*γ*-secretase-mediated cleavage of N-cadherin, which promotes *β*-catenin's nuclear translocation. Therefore, these sequential proteolytic events are considered to be a crucial step in the switch from cell-to-cell adhesion to proliferation or differentiation (Ref. 198). Ryu and co-workers have shown that *β*-catenin functions as a negative regulator of the differentiated chondrocyte phenotype. Therefore, a decrease in *β*-catenin expression is required for chondrogenic differentiation of mesenchymal cells and subsequently, a low protein abundance of *β*-catenin is necessary for the maintenance of the chondrocyte phenotype. The inhibitory role of *β*-catenin in chondrocyte differentiation is exerted by its ability to stabilise cadherin-mediated cell-to-cell adhesion, whereas loss of the chondrocyte phenotype is because of *β*-catenin's ability to regulate gene expression (Ref. 219). There is growing evidence in the literature that *β*-catenin, in general, is an important regulator of MSC lineage differentiation, and that the cellular levels and subcellular localisation of *β*-catenin, that is, nuclear versus cytoplasmic, plays a decisive role. In this context, *β*-catenin prevents adipogenic differentiation, supports the formation of early osteo-chondroprogenitor cells and its absence after this point promotes entry into the chondrocytic pathway (Refs 224–229).

## Channel-associated mechanosignalling

### Mechanoresponsive ion channels in cartilage tissue

In chondrocytes, various ion channels have been identified so far. This includes stretch-activated ion channels (SACs), voltage-gated calcium channels (VGCCs), transient receptor potential (TRP) channels, PIEZO channels, purinergic receptors and connexin-built gap junctions (GJs) and HCs (Refs 158, 178, 230–233).

In general, SACs are involved in tyrosine phosphorylation of FAK and paxillin (Ref. 158), aggrecan gene expression, reduction in MMP-3 gene expression (Ref. 234), regulation of caspases, cell proliferation (Ref. 235) and in the maintenance of the chondrocyte phenotype (Ref. 236) (Fig. 2b).

Within the cartilage channelome, calcium-activated large potassium channels (big potassium, BK, see Fig. 1c) have been identified to be activated by stretch. The shift in ion concentrations may cause instability of inducible nitric oxide synthase mRNA or increase transport of IL-4 to the extracellular fluid, both processes blocking catabolic effects.

Considering mechanoresponsive calcium ion channels, the transient receptor potential cation channel subfamily V member 4 (TRPV4) ion channel transduces mechanical loading of articular cartilage through the generation of intracellular calcium ion transients. By employing tissue-specific, inducible TRPV4 gene constructs in mice, O'Conor and co-workers could show that the loss of TRPV4-mediated cartilage mechanotransduction in mature mice reduces the severity of ageing-associated OA. However, TRPV4 loss did not alter injury-induced OA disease progression. Based on these findings, the authors conclude that different OA subtypes, namely ageing-associated and post-injury OA, may be mediated through distinct biological and mechanical mechanisms. TRPV4-mediated calcium ion signalling is a potential target for drug development and treatment in age-associated OA (Ref. 237). TRPV4 is also expressed on the chondrocytes' primary cilia, which represent highly specialised mechanoresponsive membrane compartments that regulate chondrocyte proliferation and differentiation. In this context, signalling cascades such as the canonical Hedgehog pathway are of pivotal importance (Ref. 238). On primary cilia, TRPV4 can sense osmotic pressure changes within the cartilage matrix (Refs 237, 239).

PIEZO1 and 2 are non-selective cationic channels, permeable to sodium, potassium and calcium, which are expressed in articular cartilage (Ref. 240).

Piezo proteins are the pore-forming subunits of these trimeric mechanosensitive ion channels. They open in response to stimuli such as localised membrane stretch, whole-cell poking or fluid flow, that is, shear stress. Piezo channel opening leads to Ca^2+^ influx into the cell, thereby triggering intracellular calcium signalling pathways. In a recently published study by Lee *et al*., PIEZO1 has been reported to be upregulated in IL-1*α*-stimulated porcine chondrocytes and human osteoarthritic cartilage. Furthermore, the authors could show that PIEZO1 activity resulted in increased intracellular calcium levels at baseline and in response to mechanical deformation. Consequently, elevated resting state calcium levels attenuated the dynamics of the F-actin cytoskeleton and increased mechanically induced cartilage microtrauma. Thus, the chondrocytes could be described as mechanically hypersensitive. At the transcriptional level, increased PIEZO1 expression was governed by MAP-kinase p38 and transcription factors including cellular retinol binding protein 1 (CREBP1), activating transcription factor 2 (ATF2) or hepatocyte nuclear factor 4 (HNF4) (Ref. 96). In addition to the above-mentioned channels, which regulate intracellular calcium levels, VGCCs such as L-type alpha 1C subunit (Cav1.2) have recently been shown to be indispensable for chondrogenesis during limb development in chicken or mouse embryos. Mechanistically, pharmacological inhibition by an L-type VGCC-specific blocker, or limb-specific deletion of Cav1.2, downregulates expression of genes essential for chondrocyte differentiation, including the transcription factor Sox9 as well as the ECM molecules collagen 2a1, and aggrecan, thereby disturbing regular cartilage development (Ref. 241). Although mainly sensitive to changes in the membrane potential, former studies have shown that VGCCs colocalise with mechanoperceptive *β*1 integrin adhesion complexes in mouse limb-bud chondrocytes (Ref. 242). Based on these findings, it appears plausible that VGCCs also respond to mechanical stimuli, thereby stimulating cartilage development. Of interest, it could be shown that VGCCs activity in bone and osteoblasts is critical for load-induced OA in chondrocytes. By employing both an in vivo and in vitro approach, it could be demonstrated that soluble factors related to VGCC activity, which were collected in the supernatant of osteoblasts, induced cellular hypertrophy in chondrocytes. The effect was abolished when chondrocytes were cultured in the presence of osteoblast-conditioned medium obtained from VGCCs inhibited cultures. In line with the in vitro data, T-type alpha 1H subunit (Cav3.2) VGCC null mice exhibited significantly lower articular cartilage damage compared with knees of control mice upon mechanical loading (Ref. 243). Clinical data of a recent study on osteoarthritic cartilage from patients undergoing arthroscopic knee surgery also underscore to the role of T-type VGCCs for OA development. Cav3.3 protein abundance was significantly increased here (Ref. 244).

### Connexin 43, gap junctions and hemichannels in chondrocyte mechanotransduction

Cellular communication in chondrocytes includes intercellular exchange of small RNAs, nutrients and second messengers. Although chondrocytes are spatially separated by considerable amounts of ECM within native cartilage tissue, there is compelling evidence that chondrocytes are physically connected to each other (Ref. 245). This interaction is mediated by GJs, which are generally composed of two HCs, also called connexons. Each connexon consists of six connexin subunits each. HCs allow for direct communication between the chondrocyte and its surrounding ECM. GJs containing connexin 43 (Cx43), amongst other molecules, act as metabolic regulators within chondrocytes by enabling the intercellular exchange of glucose and essential amino acids. It has been concluded that a 3D cellular network mediated through GJs may be involved in cartilage metabolic homoeostasis (Ref. 245), although not much research efforts have focused on this topic (see above).

**Fig. 3.**
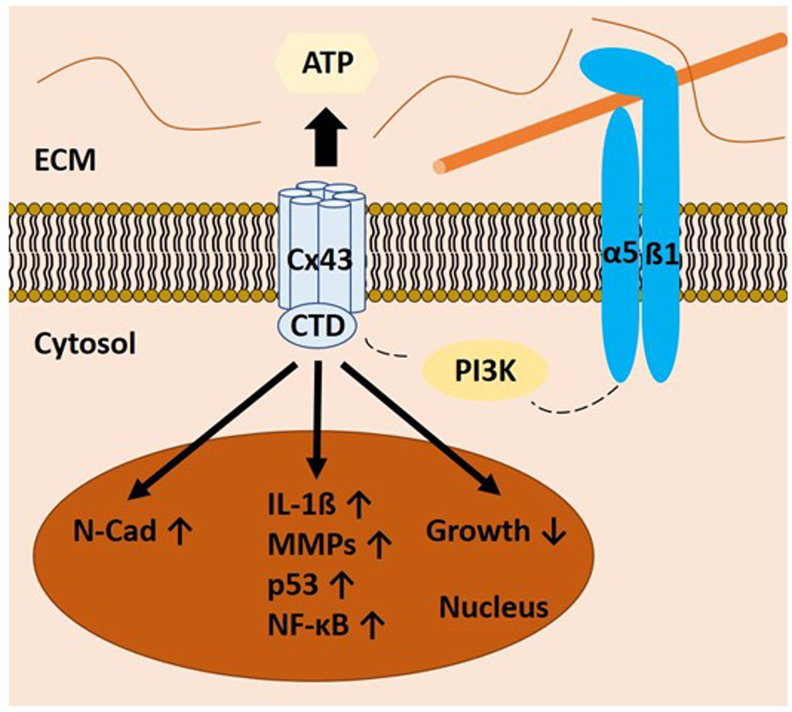
Schematic representation of Cx43-associated signalling. The C-terminal domain (CTD) of connexin 43 (Cx43) is involved in various gene regulatory processes. It can directly or indirectly upregulate proteins such as neural (N) cadherin (N-Cad), interleukin 1*β* (IL-1*β*), matrix metalloproteinases (MMPs), p53 and nuclear factor *κ*B (NF-*κ*B). Connexin hemichannels (HCs) also colocalise with *α*5*β*1 integrins. Mechanical stimulation of focal adhesions (FAs) leads to opening of the HCs via a phosphoinositide-3 kinase (PI3K)-dependent mechanism. Small metabolites such as adenosine triphosphate (ATP) can enter the extracellular space via the HCs, inducing subsequent processes such as purinergic signalling.

Cx43 is overexpressed in several human diseases and inflammatory processes including OA and IVD degeneration (Refs 246–248). The multitude of Cx43 functions is mediated through the interaction of diverse intracellular proteins with the C (carboxy)-terminal domain (CTD) of Cx43. The subcellular localisation of CTD is also relevant to its regulatory activity (Fig. 3). As an example, overexpressed CTD of Cx43 localises to the nucleus and inhibits cell growth (Refs 249, 250). Exemplarily, indirect or direct upregulation of IL-1*β*, p53, NF-*κ*B and MMPs is mediated by the CTD. Of interest, CTD also upregulates N-cadherin expression. As N-cadherin levels should decrease during chondrogenic differentiation, the Cx43 overexpression may contribute to the observed dedifferentiation of chondrocytes in OA (Refs 194, 251–254). This phenomenon is also known as chondrocyte–mesenchymal transition in the literature (Ref. 247). Herein, overactive Cx43 is causative for the maintenance of the immature chondrocyte phenotype, which, amongst others, is characterised by the synthesis of pro-inflammatory molecules including IL-1*β*, NF-*κ*B and MMPs as well as p53 (Ref. 247). These findings raise the possibility that the CTD of Cx43 can control cell cycle, gene expression or different signalling pathways independently of its channel function. Apart from this, there might also be a mechanism for a mechanosensitive *α*5*β*1 integrin-dependent opening of Cx43 HCs, which was originally described in the murine osteocyte-like cell line MLO-Y4. Integrin *α*5*β*1 interacts directly with Cx43 and this interaction, which is enhanced by fluid flow within the cartilage, is required for opening of the Cx43 HCs (see also Section ‘The ambivalent role of *α*5*β*1 integrin in chondrocyte mechano-responsiveness: cartilage maintenance versus catabolic destruction’). Direct mechanical perturbation via magnetic beads or conformational activation of integrin *α*5*β*1 also leads to the opening of the Cx43 HCs. Of interest, the integrin's function is independent of its association with an extracellular fibronectin substrate. However, phosphoinositide-3 kinase signalling is needed for the mechanically induced conformational activation of integrin *α*5*β*1 leading to the opening of the HCs. It is tempting to speculate that this cascade could be a potential mechanistic link between mechanical overload, the ambivalent role of *α*5*β*1 integrin signalling in different mechanical contexts and Cx43 overactivity (Ref. 255). If, for example, the Cx43 overexpression would be an early event during the onset OA, *α*5*β*1 integrin activation could lead to the opening of more Cx43. Then, some of the effects described in the context of the ‘overload’ response of *α*5*β*1 integrin in Section ‘The ambivalent role of *α*5*β*1 integrin in chondrocyte mechano-responsiveness: cartilage maintenance versus catabolic destruction’ could actually be attributed to the Cx43 overactivity. This thesis is supported by the fact that the downstream effects of Cx43 overactivity and the response of *α*5*β*1 to non-physiological mechanical stimuli are similar (e.g. the expression of MMPs and pro-inflammatory cytokines). Inhibiting Cx43 function could, thus, potentially lead to a restoration of proper *α*5*β*1 integrin function (see below).

HC opening additionally enables adenosine triphosphate (ATP) release from the cell and thereby facilitates ATP interaction with cellular purinergic receptors, for example, P2X and P2Y (for review, see, Ref. 256). P2X is a cation channel itself. Activation of this protein causes alterations in cellular ion concentrations. P2Y is a G protein-coupled transmembrane receptor. P2Y- and P2X-associated signalling cascades further influence the chondrocytes’ inner milieu.

## The role of the actin cytoskeleton in chondrogenesis

### Rhoa and ROCK, Rac1 and Cdc42 and their role in cartilage mechanotransduction

Cell–matrix contacts via integrins and cell-to-cell contacts via cadherins intracellularly address the constituents of the actin cytoskeleton. Thus, the actin cytoskeleton is the common final route of the mechanotransduction pathways presented in Sections ‘Mechanotransduction at the cell–matrix interface’ and ‘Mechanotransduction at the cell–cell interface’. Above, the intracellular ion concentrations, especially that of calcium, tremendously influence the dynamics of the acto–myosin system. This is an important connection of the function of mechanosensitive ion channels and the cytoskeleton (Ref. 257).

Actin is an important structural protein within cells and is amongst the most abundant proteins in eukaryotes. In its monomeric form, called globular (G)-actin, it binds ATP. Upon polymerisation into filamentous (F)-actin, ATP is hydrolysed. Dynamic de- and re-polarisation of actin as well as its crosslinking or anchoring at the plasma membrane and organelles enables fast adaptation of cellular morphology and the initiation and maintenance of processes such as cell migration. Motor proteins from the myosin family, which are associated with F-actin, also allow for cell contractility (e.g. during mitosis) and transport of molecules and organelles along the actin filament system (Ref. 257).

For actin reorganisation, the small GTPases ras homologue family member A (RhoA), Ras-related C3 botulinum toxin substrate 1 (Rac1) and cell division control protein 42 homologue (Cdc42) are essential (Refs 258–261). They regulate cell shape and cytoskeletal tension. GTPases act as molecular switches. This means that they exert their function only if guanosine triphosphate (GTP) is bound to the protein. Upon GTP hydrolysis to guanosine diphosphate, the proteins lose their regulatory activity. RhoA is especially important for the formation of actin stress fibres, and it promotes contractility of the actin cytoskeleton. The Rho-associated protein kinase (ROCK) is a downstream effector of RhoA and is a key regulator of cell migration and myosin-dependent cell contractility. Above, it inhibits actin depolymerisation. Rac1 activity is needed for the building of lamellipodia. Cdc42 is involved in cell cycle progression, endocytosis and cell motility (Refs 258–261).

**Fig. 4.**
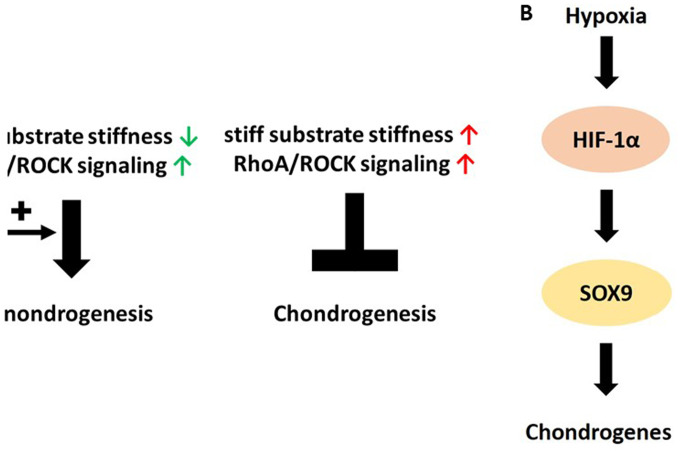
Illustration of the actin-associated signalling in chondrogenesis. (a) Influence of hypoxia, substrate stiffness and ras homologue family member A (RhoA)/RhoA-associated protein kinase (ROCK) signalling on chondrogenesis. Details are given in the main text. (b) Chondrogenic signalling cascade associated with hypoxia. Details are given in the main text. HIF-1*α*, Hypoxia-inducible factor 1*α*; Sox9, SRY-box transcription factor 9.

With respect to the molecular basis of chondrogenesis, cytoskeletal tension appears to be a central point. Cytoskeletal tension is also related to the cell's morphology. This tension is regulated by a pathway involving RhoA and ROCK, which have been identified as direct regulators of chondrogenesis (Ref. 90). Whether RhoA/ROCK acts as pro- or anti-chondrogenic depends on the direct cellular context and/or the cells' environmental stiffness. These complex interplays are described in the following: adipocytes and chondrocytes are both derivatives of MSCs and exhibit roundish cell morphologies (Ref. 89). Adipogenesis and mesenchymal condensation during chondrogenesis both take place in an extracellular environment of soft stiffness and the cells adopt to a roundish morphology. Contrary to adipogenesis, where cytoskeletal tension is low, chondrogenesis needs cytoskeletal tension as a driver of mesenchymal condensation (Refs 262, 263). Hence, in the case of soft substrate stiffness, high RhoA/ROCK activity is important for chondrogenesis. On stiff substrates, where cells adopt a flattened shape, RhoA-mediated ROCK activity counteracts chondrogenesis (Fig. 4a). With respect to substrate stiffness, Allen and co-workers demonstrated that RhoA/ROCK-inhibited ATDC5 cells (isolated from the differentiating teratocarcinoma stem cell line AT805, which is commonly used as a model for in vitro chondrogenesis (Ref. 197)) at the early stages of differentiation exhibit a downregulation of chondrogenic biomarkers on soft substrates. Contrary to that, inhibition of ROCK yielded biomarker upregulation, including SOX9, collagen type II alpha 1 and aggrecan on stiff substrates (Ref. 264).

Experiments in two-dimensional (2D) monolayer cultures and 3D-micromass cultures with MSCs and the ATDC5 cell line, however, challenge the overall applicability of the theory on cytoskeletal tension and chondrogenesis. Woods and Beier could demonstrate that the ATDC5 cells in conventional monolayer cultures exhibited a progressive decrease in RhoA activity during chondrogenic differentiation, concomitant with increased expression in collagen type II and aggrecan. Overexpression of RhoA reduced the expression of cartilage developmental markers. In micromass cultures, which more adequately represent the spatial distribution and interaction of cells during chondrogenesis, RhoA/ROCK inhibition led to an increase in SOX9 expression. The SOX9 target genes collagen II and aggrecan, however, did not respond to this increase in SOX9 protein. The latter finding is in accordance with the above-described theory that RhoA/ROCK activity is needed for chondrogenesis on soft substrates. Thus, the fine-tuning of RhoA/ROCK contribution to chondrogenesis depends on the exact cellular context, for example, whether studies are performed in 2D monolayer cultures or 3D micromass cultures (Ref. 265). Moreover, the same research group could show in a previous study that RhoA/ROCK-signalling is important to maintain proliferation and to suppress hypertrophic chondrocyte differentiation in ATDC5 cells as well as in primary chondrocytes. This was indicated by a marked delay in the onset of collagen X and MMP-13 (both markers or chondrocyte hypertrophy) gene expression in the absence of ROCK inhibition or while overexpressing RhoA (Ref. 266).

RhoA/ROCK activity is, however, not the only determinant of chondrogenesis. Chondrocyte-specific gene expression, including SOX9, collagen II and aggrecan, can be increased, if the cells are not cultured under normoxic but hypoxic conditions. Hypoxia is physiological to chondrocytes in vivo because cartilage is avascular and oxygen only reaches the tissue via diffusion. This suggests a synergism of Rho-kinase signalling and hypoxia on chondrocyte-specific gene expression (Ref. 267). At the molecular level, hypoxia upregulates hypoxia inducible factor (HIF), which in turn stimulates SOX9 expression. The latter is essential for chondrogenesis and maintains the articular chondrocyte phenotype throughout its lifespan (Refs 268–270) (see Fig. 4a and b and Ref. 271).

In addition to RhoA/ROCK, Rac1 and Cdc42 also participate in actin cytoskeleton reorganisation. Activation of these small GTPases is required for many biological processes such as cell spreading, motility and phagocytosis (Refs 272, 273). In the cartilage context, it could be shown that both Rac1 and Cdc42 are essential for chondrocyte condensation and N-cadherin expression, thereby promoting chondrogenesis (Ref. 274). A recent study on micromass cultures of chick wing bud mesenchyme could show that Rac1 additionally promotes chondrogenesis via BMP4. BMP4 expression was guided by Rac1-mediated phosphorylation and activation of signal transducer and activator of transcription 3 (STAT3). STAT3 activation requires increased expression of IL-6, which is also regulated by Rac1 (Ref. 275). Besides their role in chondrogenesis, Rac1 and Cdc42 have been demonstrated to support chondrocyte differentiation. Overexpression of Rac1 and Cdc42 in ATDC5 cells accelerated hypertrophic differentiation and chondrocyte apoptosis. In primary chondrocytes, Rac1 and Cdc42 push the collagen type X promoter to its maximum activity, an effect mediated by the activity of the MAP kinase p38 (Ref. 276). Moreover, Rac1 promotes expression of the hypertrophy-indicating biomarker MMP-13 during growth plate differentiation. This process has been shown to be mediated by fibronectin fragments in vitro (Ref. 277). Because hypertrophic chondrocytes expressing collagen type X and MMP-13 are also found in OA and IVD degeneration, the pathogenic contribution of Rac1 might yet be underestimated (Refs 278, 279).

## Perspectives in cartilage regeneration

### Integrins, connexins and mechanoresponsive ion channels

Tissue regeneration strategies often make use of stem cells. Therefore, cell-instructive materials (e.g. hydrogels) in the context of cartilage biology should be able to guide the spatiotemporal development from MSCs or CPCs to mature cartilage. Mechanotransduction-inspired biomaterials for cartilage regeneration would benefit from including functional groups (e.g. specific peptide sequences such as the RGD or the HAVDI motif) that are able to interact with the above-described mechanotransduction pathways in a specific manner. This would both lead to a deeper understanding of the mechanism of biomechanical tissue homoeostasis and allow for targeted, pathway-specific induction of biological responses. The overall aims are to generate a microenvironment that (i) guarantees an anti-inflammatory tissue phenotype (i.e. suppression of pro-inflammatory cytokines such as IL-1*β* or TNF-*α*), (ii) enables the synthesis of ECM components of mature cartilage (i.e. collagen type II, aggrecan and cartilage oligomeric matrix protein), (iii) supports the differentiation of cartilage (i.e. induction and maintenance of SOX9) and (iv) inhibits the production of matrix-degrading factors (i.e. MMPs).

These points are all at least, in part, mediated by the integrin system. It is technically possible to couple nearly any naturally occurring ECM ligand to a synthetic hydrogel-polymer, for example, by coupling fibronectin domains or peptides to a PEG backbone to specifically stimulate *α*5*β*1-integrin, which has been shown to be a key player in chondrocyte mechanobiology and regeneration (Ref. 146). Especially, the RGD-tripeptide (arginine–glycine–aspartate) of fibronectin is required for integrin binding. However, one major obstacle remains the still insufficiently understood role of fibronectin fragments in cartilage biology. Some authors reported that these fragments promote ECM remodelling and have cell-protective effects on chondrocytes (Refs 209, 280). Contrary to that, others showed that fibronectin fragments promote the progression of OA by stimulating the expression of MMPs and catabolic cytokines (Refs 175, 187, 281).

Therefore, it is of great importance to further clarify the effect of integrin ligands on human chondrocyte biology, which also includes ligand concentration effects and the exact force-dependency of integrin–ligand interactions and their consecutive downstream signalling.

Apart from that, it is tempting to experiment with modified or completely synthetic integrin ligands. This approach could potentially reduce the biological complexity and context-dependency of the above-described signalling processes. However, it would also increase the financial and regulatory obstacles connected to translation. Exemplarily, Almonte-Becerril *et al*. have developed a mouse model with mutated RGE (arginine–glycine–glutamic acid) amino acid-motif containing fibronectin (Ref. 282). Interestingly, these mice had normal bone and cartilage tissues and, in contrary to wild-type mice, moderate mechanical stress did not lead to cartilage destruction despite the elevated levels of MMP-3 and MMP-13. This indicates that simple modification of amino acid binding motifs has the potential to select between intended and off-target effects of ligand–receptor interactions. This knowledge could, therefore, be transferred to innovative biomaterials.

Apart from that, it is of great importance to consider the developmental stage of the cells that are used for regenerative purposes. This is necessary because clinical tissue engineering applications either use autologous donor cells, for example, MSCs, which are re-transferred to the patient within a biomaterial scaffold or use biomaterials to stimulate the patient's chondrocytes *in situ*. Thus, regeneration strategies that make use of CPCs or MSCs should actively stimulate chondrogenic lineage differentiation. This is because both cell types are still in a state of developmental plasticity and their differentiation depends on adequate external and internal signals. Interaction with *in situ* chondrocytes should, however, foremostly aim at maintaining the chondrocyte phenotype because differentiation has already taken place.

If a biomaterial is transferred into the patient, it must be regarded as a ‘substitute’ ECM. As these biomaterials can be constructed from naturally occurring ECM ligands, are hybrid or fully synthetic, proper composition of this ‘substitute’ ECM is a key challenge. Thus, molecules such as the two below-presented examples that strongly support chondrogenesis are promising candidates for stem cell-based therapies in cartilage biology (Ref. 283). Laminin-*α*1, recognised by *α*3*β*1 integrin, enhances the expression of articular cartilage collagen type II, and restricts collagen type I abundance, which is a marker of chondrocyte degeneration and cartilage degeneration in the context of OA. In addition, nidogen-2, another cartilage ECM constituent, leads to an increase in SOX9, a core transcription factor in cartilage biology, and counteracts RUNX2 expression, which is needed for osteoblast differentiation.

Moreover, ex vivo biophysical stimulation of cells that are going to be transferred into a patient may be beneficial in the treatment of cartilage degeneration (so-called ‘loading history’ of the cells). Salter and co-workers showed that this approach led to, for example, downregulation of MMPs, thereby reducing catabolic effects (Ref. 284). However, this also increases the obstacles related to translation.

Another strategy to simultaneously favour both cartilage formation and OA prevention may arise from the findings published by Song *et al*. The authors demonstrated that the integrin inhibitor ten beta integrin epidermal growth factor-like repeat domains (ITGBL1) reduces integrin signalling through the prevention of integrin–ECM interactions. The latter is critical for cartilage formation and development of OA. Regarding cartilage formation, ITGBL1 is expressed in developing chondrocytes at specific stages and it is downregulated in the case of OA (Ref. 285). Thus, incorporation of integrin inhibitors, such as ITGBL1 or corresponding synthetic mimics, into future biomaterial concepts could be promising.

As overexpression of proteins such as Cx43 is an important marker of OA-affected cartilage, it is tempting to address this target in regenerative strategies. It is known from other fields of biomedical research that the inhibition of Cx43 can protect cells and tissues from damage, as demonstrated, for example, in acute kidney injury (Ref. 286). In this study, the connexin decoupling agent heptanol was used to reduce connexin-related signalling such as the initiation of pro-inflammatory pathways including, for example, IL-1*β*. Many other connexin-inhibiting agents have been described so far, amongst others Gap19 and carbenoxolone (Refs 287, 288). The latter was used in an in vitro study employing chondrocytes. It could be shown that the inhibition of Cx43 led to a redifferentiation of osteoarthritic chondrocytes to the mature phenotype. Above, cellular senescence and pro-inflammatory mediators such as IL-1*β* and the catabolic MMPs were attenuated upon the inhibition. These two examples show that Cx43 is a core pathogenic factor in many tissues and its manipulation is a promising target in OA treatment. However, when addressing Cx43 by cell instructive biomaterials, it is vital to respect their interactions and effects on typical chondrocyte integrins, N-cadherin in conjunction with *β*-catenin, and N-CAM, which were explained above. N-Cadherin is a transcriptional target of the CTD of Cx43. However, strong N-cadherin expression is only needed during precartilage condensation. Additionally, the signalling crosstalk between Cx43 and *α*5*β*1 needs to be considered. A hydrogel that is, for example, extensively interacting with *α*5*β*1 could overstimulate the Cx43-related signalling pathway as an off-target effect, which could promote a pro-inflammatory milieu and inhibit chondrocyte differentiation. Consequently, a promising approach would be to combine integrin stimulation with connexin inhibition. By applying this principle, it would be possible to use the anabolic, anti-inflammatory consequences of *α*5*β*1 signalling during physiological loading by simultaneously blocking the catabolic effects of Cx43 overexpression. Several hydrogels with different modes of drug release have been presented so far, as exemplified in Ref. 289. Non-toxic connexin inhibitors such as Gap19 could then be incorporated into a mechanobiology-inspired, cell instructive biomaterial with, for example, RGD peptides, leading to maintained integrin signalling and an ‘understimulation’ of Cx43. A prerequisite for this approach is the determination of proper drug release constants and drug concentrations and the quantitative influence of mechanical loading and the degree of integrin stimulation on the interaction with connexins and their inhibitors.

Regarding mechanoresponsive ion channels, it is also important to consider the appropriate amount of mechanical stimulation at the correct point of time. Because knockout of TRPV4 can stop the cascade associated with age-related OA, it is tempting to speculate that its inhibition is a good target for clinical OA disease mitigation. Although many so-called disease-modifying OA drugs are in clinical development at the moment, TRPV4 inhibitors have not yet been tested for OA in patients (Ref. 290). A recent study employing GSK2798745, a TRPV4-inhibitor, in heart failure patients did not raise any significant safety concerns. These promising results raise hope for future application of this type of drugs in OA (Ref. 291). The activity of mechanosensitive ion channels such as PIEZO1 could be addressed by the development of drugs directed against MAP-kinases such as p38 or transcription factors such as CREBP1, ATF2 or HNF4, because these molecules are involved in increased PIEZO1 expression during OA (Ref. 96). In vitro studies using human chondrocytes and bone marrow-derived mesenchymal stem cells (BMMSCs) showed that the antihypertensive drug nifedipine, which blocks L-type VGCCs by hindering Ca^2+^ influx into the cell, exhibited positive effects on the synthesis of collagen type II and proteoglycans in both cell types. This implies that this drug exerts beneficial anabolic responses in articular cartilage in the context of VGCC signalling (Ref. 292).

### Cadherins

N-Cadherin-dependent signalling events, that is, the initiation of mesenchymal cell condensation and the related mechanosignalling, are a promising target, which can be addressed to initiate the cascade of cartilage formation. As described above, the cell–cell adhesion via cadherins is only needed at the very beginning of precartilage condensation, whereas the degradation of N-cadherin and the *β*-catenin-related signalling events are subsequently needed for a switch to proliferation and differentiation. Hence, as discussed in Section ‘Cadherins and their role in cartilage mechanotransduction’, the sequential presentation of cell–cell adhesion and cell–ECM interaction in an engineered microenvironment seems to be a promising strategy to facilitate mesenchymal condensation and chondrogenic differentiation (Ref. 73).

Innovative regenerative biomaterials may, therefore, provide (i) N-cadherin domains or peptides that initially stimulate MSCs to condense, and (ii) an inherent mechanism that allows these N-cadherin mimetics to be degraded to initiate the phase of proliferation and differentiation. Guo *et al*. described modular and biodegradable cross-linkers for hydrogels, for example, PdBT with parts of the N-cadherin ectodomain, and they can be modified in a way that the degradation constants properly fit the natural kinetics of cartilage development (Ref. 146). It has been recently shown that nanofibre systems composed of lauryl–peptide conjugates with the conserved HAV (His-Ala-Val) motif of N-cadherin can efficiently induce cell–cell adhesion of MSCs (Ref. 293). A similar approach was applied in an aggrecanase-1 degradable hydrogel in a rabbit model, which led to better regeneration results (Ref. 294).

Based on their spatial and functional discrimination, integrins and cadherins are often treated independently in the context of mechanosignalling. However, intracellular signalling crosstalk leads to a tight interrelationship and interdependency of both contact sites. This can be understood by the joint usage of downstream signalling proteins by both cell–cell (i.e. cadherin-based) and cell–matrix (i.e. integrin-based) contacts. The most obvious common final route of these mechanotransduction cascades is the cytoskeleton, whose functional state consequently governs cell behaviour. This interplay can be exemplified by the activity of the small GTPase Rac1 (Section ‘Rhoa and ROCK, Rac1 and Cdc42 and their role in cartilage mechanotransduction’). Rac1 activation boosts proliferation by upregulating cyclin D1 expression, regardless of being activated by either cadherins or integrins (Ref. 295). Furthermore, it has been reported that N-cadherin in cooperation with the fibronectin receptor (*α*5*β*1 integrin) is involved in hMSCs lineage determination through Rac1 (Ref. 296).

Therefore, the next step in regenerative strategies is to address the interplay of integrin and cadherin systems as well as the signalling of ion channels, which could provide a method for induction (e.g. cadherin-dependent mesenchymal cell condensation) and maintenance (e.g. *α*5*β*1 integrin- and Cx43-dependent ECM synthesis) of chondrocyte phenotype. A recent in vitro study has demonstrated the role of biophysical cues in integrin-FAK-related mechanotransduction and its consequences on *β*-catenin in BMMSCs and primary chondrocytes. The authors could show triggering of the osteogenic phenotype of BMMSCs and primary chondrocyte phenotype maintenance on polyacrylamide gels of increasing stiffness, which were coated with collagen type I and collagen type II. At the mechanistic level, the cellular mechanoresponsive behaviour involved the activation of *α*1*β*1 and *α*10*β*1 integrin–FAK pathways, which led to phosphorylation-dependent inactivation of GSK3*β* (glycogen synthase kinase 3 beta) (Refs 297–300). Consequently, *β*-catenin accumulated both in the cytoplasm and the nucleus in a Wnt-independent manner. *β*-Catenin within the nucleus in turn bound to the Wnt1 promoter region to up-regulate gene transcription supporting the chondrocyte phenotype at a matrix stiffness of around 0.1 MPa (Ref. 298). These findings underscore the mechanistic interplay of mechanotransduction pathways in post-membranous signalling. The understanding of these synergistic signalling effects on chondrocyte biology may be used to optimise regeneration strategies for cartilage defects, as they offer a unique combination of spatiotemporal regulation of condensation, proliferation and differentiation.

### The cytoskeleton

Substrates for regenerative strategies employing pre-chondroblasts, that is, cells determined for the chondrocyte lineage, or cells just undergoing chondroblast differentiation should promote a proper cellular morphology. As described in Section ‘Rhoa and ROCK, Rac1 and Cdc42 and their role in cartilage mechanotransduction’, cells in this developmental stage exhibit roundish shapes. The morphogenesis is supported by a soft substrate stiffness and a high RhoA/Rock-mediated cytoskeletal tension, as recently discussed in Ref. 9. Strategies that utilise mature chondrocytes would correspondingly benefit from low levels of RhoA/ROCK activity (Ref. 301). In combination with substrate stiffness, biochemically simulated hypoxia, which addresses SOX9 via the HIF pathway, appears to be a promising synergistic parameter to be included in future mechanotransduction-based cartilage regeneration strategies (Ref. 268). Hypoxia signalling pathways also inhibit the expression of collagen type X, which is the major marker for chondrocyte hypertrophy and subsequent calcification. Hypoxia-related signalling could thus prevent the potential calcification of engineered cartilage and might be a useful tool in treatment concepts, where chondrocyte hypertrophy and subsequent bone formation must be avoided.

Here, again hydrogels may be a promising concept for an HIF-1*α*-supplemented biomaterial-based strategy. Because many biomaterials are composed of viscous polymers made of synthetic or natural hydrophilic macromolecules, which are able to form gels after physical, ionic or covalent crosslinking (Refs 302–304), they could be used as carriers for bioactive molecules, that is, HIF-1*α* modified with cell-penetrating peptides (CPPs) (Ref. 305). These peptides enable the delivery of the transcription factor to the interior of the cell so that it can exert its functions in gene regulation. This treatment concept would enhance the physiological hypoxia in the articular environment by additionally and artificially triggering hypoxia signalling pathways via the CPPs. One advantage of this strategy is that the hypoxic articular conditions can already be simulated ex vivo and the amount of HIF-dependent signalling can be determined independent from oxygen partial pressure. Although scientifically exciting, the regulatory perspectives of such approaches are not very promising because delivery of proteinaceous therapeutics is strictly regulated.

Concerning cytoskeleton regulating proteins, the same principles as for influencing connexin signalling or HIF-1*α* can theoretically be applied. Calpeptin is an example of a well-known activator of RhoA function (Ref. 306). Together with an appropriate material stiffness and the delivery of HIF-1*α*, controlling RhoA signalling seems possible. Again, it is important to consider degradation and diffusion constants of the respective materials.

As discussed in Section ‘Rhoa and ROCK, Rac1 and Cdc42 and their role in cartilage mechanotransduction’, Cdc42 and Rac1 are important for both chondrogenesis as well as progression of chondrocyte differentiation up to hypertrophy. Thus, in favour of cartilage regeneration, appropriate strategies should address their activation during early chondrogenesis, while subsequently prompting their suppression to avoid hypertrophy. Regarding such strategies, a promising approach emerged from the application of the Rac1 inhibitor NSC23766. This low molecular-weight inhibitor was incorporated into chitosan microspheres. These microspheres served as a delivery vehicle for the drug to the site of action, that is, the knee joints of mice. There it efficiently protected cartilage from destruction (Ref. 307).

## Conclusions and open research questions

The central research question in regenerative cartilage biology is how to reproduce the in vivo processes of cartilage development and maintenance to mitigate or cure diseases of this tissue. This includes the formation of the right type of cartilage in the right place at the right time. More specifically, we were interested in the current scientific evidence for the involvement of major mechanotransduction pathways in cartilage biology and pathophysiology.

In vivo chondrogenesis after injury often leads to fibrocartilage formation (e.g. when applying ACI), whereas hypertrophic cartilage is often the result of in vitro chondrogenic differentiation of MSCs. As the current medical treatment options for OA and IVD degeneration show, the development of a successful therapy that is governed by the molecular mechanisms of cartilage biology is hard to accomplish. This might be related to the fact that the mechanisms behind the formation of proper hyaline cartilage rather than fibrocartilage or hypertrophic cartilage are still incompletely understood. Translation of innovative approaches to clinical treatments is also hindered by the complex and financially demanding approval process.

Current surgical treatment paradigms range from microfracture surgery for relatively small cartilage defects to total joint replacement in advanced OA. IVD treatment is foremostly based on physical exercises, pain management or surgical decompression/stabilisation of the affected vertebral segments. However, there is no universal agreement on solutions in bioengineering that have unequivocally been shown to fully regenerate structural and functional cartilage (Ref. 308). Current trends in the fabrication of biomaterials for cartilage repair are focused on material modifications for improved control of the biochemical and biomechanical milieu, specifically for the application in challenging situations such as in early/moderate OA, as the many examples in the main text have shown (Refs 142, 143).

However, until now, no mechanosensing and thus mechanotransduction-based biomaterial concept has been established experimentally or even reached the clinic. Future research for advanced biomaterial development for articular cartilage regeneration would benefit from focusing on these strategies to provide a tissue-innate, regeneration-supporting biomechanical environment.

In this review, we have summarised important knowledge on the role of special integrins, cadherins and ion channels for mechanotransduction in cartilage biology and pathophysiology. We furthermore introduced current research that is based on the cell-physiological functions that are described herein and discussed open research questions. Ideas for further improvements of these strategies by incorporating biomechanical concepts were explained as well. Altogether, we have shown how the integration of biophysical cues in the context of integrin-, cadherin-, channel- and cytoskeletal signalling may be valuable for constructing advanced regeneration-inducing biomaterials. Such ‘next-generation’ biomaterials would possess cell-instructive properties, which would, for example, aim at guiding MSCs in their development from cellular condensation to proliferation and differentiation. This could finally enable high-quality articular cartilage regeneration. From a regulatory perspective, these types of biomaterials, containing stem cells, extensive protein modifications or drug delivery mechanisms, will be considered as ‘advanced therapy medicinal product (ATMP)’. This type of medical device will be subjected to the regulatory approval process as a drug including subsequent time-consuming and expensive clinical trials. However, the potential benefits would likely outweigh the enormous costs. Thus, research efforts would benefit from focusing on integrating selected, promising aspects of the complex signalling processes of chondrocytes and MSCs and utilising specific interactions with natural and synthetic environments to achieve high-quality regeneration via cell-instructive biomaterials. As discussed in Section ‘Biomaterials and the concept of cell instruction’, a promising approach is represented by the trilayered hydrogel scaffold presented by Kang and co-workers (Ref. 147), which appears to be cell-instructive for both MSCs and chondrocytes. Because of its specific configuration, it enables the formation of cartilage and bone tissue. In this context, an open question refers to the mechanical properties of biomaterials to be used for cartilage regeneration. Naturally occurring biomaterials are known to lack the mechanical resilience of mature articular cartilage, hindering their clinical translation. Thus, the research in this field is shifting towards the mechanical tuning of natural or synthetic biomaterials or the development of new IPN, the latter discussed in Section ‘Biomaterials and the concept of cell instruction’.

Mechanobiology-related mechanotransduction is, of course, only one cornerstone in this process. But, it offers plentiful possibilities to improve regenerative strategies that will ultimately be effective. Hopefully, the concepts presented within this review will in the future be integrated into clinically applicable therapy concepts to lessen the morbidity associated with cartilage- and joint-associated diseases.
